# The effect of melt-spun phase change material fibre garments on skin temperature in humans

**DOI:** 10.1186/2046-7648-4-S1-A75

**Published:** 2015-09-14

**Authors:** Maria Suong Tjønnås, Hilde Færevik, Mariann Sandsund, Randi E Reinertsen

**Affiliations:** 1 SINTEF Technology and Society, department of Health Research, Trondheim, Norway

## Introduction

Phase change materials (PCM) have the ability to store latent heat, a property that is utilized in the making of thermo regulating clothing [1]. The melt-spun PCM fibre [2] has a polyester sheath and PCM filled core, giving the fibre its cooling properties. By virtue of being a fibre, it enables production of a knitted fabric with high air and moisture permeability as well as high flexibility qualities. The flexible knitted fabric allows for better-fitting clothes [3], and larger area of the body surface to be covered and exposed to the cooling effects of the melt-spun PCM fibre. The aim of this study was to investigate the effects of a melt-spun PCM fibre sweater with PCM melting temperature at 28.4 °C and crystallisation temperature at 25.2 °C, on the skin temperatures of the upper body and perceived thermal sensation and comfort in a hot environment.

## Methods

Fourteen test subjects (8 male, 6 female; mean (SD) age 23 (3) y; height 171 (11) cm; weight 65.3 (8) kg) completed a passive heat session (rested on a chair in a climatic chamber at 30 °C and 50 % rh). Skin thermistors were placed on 4 different locations, the upper arm, chest, scapula and lower back to measure the average skin temperature of the upper body. When the skin temperatures were stabilised, the cooling PCM fibre sweater (estimated PCM mass; 28 (g), specific latent heat capacity; 1.842(kJ)) was put on in an ambient temperature of 30.5 (0.5) °C to ensure that the PCM would be in a liquid phase. The test subjects sat for 20 minutes further in the climatic chamber. The perceived thermal sensation and comfort was rated by each test subject at particular points during the test course. The same protocol was repeated with a sweater made up of 100 % polyester, the same polyester fibres as in the PCM sweater without any PCM. This polyester sweater served as a reference sweater to the PCM fibre sweater in this experiment.

## Results

The average skin temperatures were 0.3 - 0.5 °C lower when wearing the PCM fibre sweater compared to the reference sweater, (P < 0.05). This cooling effect on the skin was first recorded one minute after the test subjects had put on the PCM fibre sweater, and lasted for 8 minutes following. No significant differences in the perceived thermal sensation and comfort were seen between the PCM fibre sweater and the reference sweater.

## Discussion

The latent energy capacity was large enough to generate a cooling effect which could be recorded for 8 minutes, but not large enough to have an impact on the perceived thermal sensation or comfort. The amount of PCM mass incorporated in the fibres was likely too small to generate a long lasting cooling effect [4].

## Conclusion

Although the flexible PCM fabric covered a large area of the body surface, the cooling effect of the melt-spun PCM fibre was relatively short-lasting. More PCM mass incorporated in the fibre may provide a longer lasting cooling effect on the skin temperature of the upper body, and affect the perceived thermal sensation and comfort, and consequently, production of better temperature regulating clothing.
